# Interpretable Machine Learning Enables Preoperative Physiologic Risk Stratification for Dysphagia After Anti-Reflux Surgery

**DOI:** 10.21203/rs.3.rs-10250026/v1

**Published:** 2026-07-14

**Authors:** Anjani H Turaga, Yashwanth Alakky, Paneed Jalili, Anne Worth, Brenden M Finnerty, Thomas J Fahey, Rasa Zarnegar

**Affiliations:** Weill Cornell Medicine; Cornell University; Weill Cornell Medicine; Weill Cornell Medicine; Weill Cornell Medicine; Weill Cornell Medicine; Weill Cornell Medicine

## Abstract

Postoperative dysphagia remains one of the most clinically significant complications following anti-reflux surgery, yet existing preoperative risk stratification approaches incompletely integrate esophageal motility, reflux burden, and esophagogastric junction biomechanics. We developed an interpretable machine learning framework integrating multimodal physiologic and clinical data to predict new-onset postoperative dysphagia and translate these relationships into a clinically deployable risk score. Following screening of 878 consecutive patients undergoing anti-reflux surgery at a high-volume tertiary referral center, 428 patients met inclusion criteria and underwent multimodal preoperative physiologic assessment including high-resolution manometry, EndoFLIP impedance planimetry, Bravo pH monitoring, and GERD-HRQL evaluation. Patients were divided into derivation (n = 362) and independent holdout validation (n = 66) cohorts. An ensemble machine learning framework integrating logistic regression, random forest, XGBoost, and support vector machine models was developed using engineered higher-order physiologic interaction features and subsequently translated into an interpretable point-based scoring system.

The ensemble model demonstrated strong discrimination in the derivation cohort (AUC = 0.91, accuracy = 80.9%, sensitivity = 86.2%, specificity = 75.7%) with preserved performance in the independent holdout cohort (AUC = 0.72, balanced accuracy = 68.7%, sensitivity = 70.0%, specificity = 67.4%). Interaction features integrating reflux burden, esophageal contractility, distensibility, and patient-level modifiers demonstrated greater predictive utility than isolated physiologic variables alone. The resulting cumulative risk score enabled stratification into distinct postoperative dysphagia susceptibility groups across both derivation and validation cohorts and was deployed as an open-source web-based calculator for individualized risk estimation.

These findings demonstrate that integrated multimodal esophageal physiology combined with interpretable machine learning enables clinically meaningful prediction of postoperative dysphagia after anti-reflux surgery and may support future personalized perioperative risk stratification and prospective multicenter validation.

## Introduction

Dysphagia remains one of the most common and clinically consequential complications following anti-reflux surgery. Although transient swallowing difficulty is expected in the immediate postoperative period due to edema and altered esophagogastric junction (EGJ) mechanics, a subset of patients develops persistent dysphagia that significantly impacts quality of life, dietary tolerance, and overall satisfaction with surgery [1, 2]. Importantly, this complication is often difficult to predict preoperatively, limiting the ability of surgeons to counsel patients effectively or tailor operative strategy.

The pathophysiology of postoperative dysphagia is multifactorial, involving a complex interplay between esophageal motility, EGJ compliance, reflux burden, and technical aspects of the operation [3, 4]. Conventional preoperative assessments including high-resolution manometry, ambulatory reflux monitoring, and endoscopic evaluation provide valuable but fragmented insights into these domains. However, these modalities are typically interpreted in isolation, and their individual predictive value for postoperative dysphagia has been inconsistent across studies [5, 6]. As a result, current clinical decision-making relies heavily on surgeon experience and heuristic interpretation rather than objective, integrative risk stratification.

Historically, identifying patients at risk of postoperative dysphagia would require large, prospective, multi-institutional studies with standardized physiologic and clinical data collection. While such trials have provided important insights into surgical outcomes, they are resource-intensive and often impractical for capturing the complex interactions between physiologic variables that influence postoperative function [7]. Consequently, despite decades of investigation, there remains no widely accepted preoperative predictive framework for dysphagia following anti-reflux surgery.

Advances in machine learning offer a fundamentally different approach to this problem. By enabling the simultaneous integration of high-dimensional clinical, physiologic, and demographic data, machine learning models can capture nonlinear relationships and interactions that are not readily apparent using traditional statistical methods [8, 9]. In gastroenterology and esophageal disease, machine learning has already demonstrated utility in areas such as motility pattern recognition and diagnostic classification, highlighting its potential to augment clinical decision-making [10, 11].

In this study, we sought to leverage machine learning to address this unmet clinical need. Our primary objective was to develop and internally validate a predictive model for new-onset dysphagia following anti-reflux surgery using preoperative clinical and physiologic variables. Our secondary objective was to translate this model into an interpretable, point-based risk score that could be applied in routine clinical practice. By integrating multimodal data into a unified predictive framework, we aimed to move beyond isolated physiologic metrics and toward actionable, preoperative risk stratification.

## Results

### Study population and baseline characteristics

A total of 878 patients who underwent anti-reflux surgery were screened for inclusion. After applying predefined inclusion criteria, 362 patients were included in the derivation cohort and 66 patients in the independent validation cohort.

Baseline demographic, clinical, and physiologic characteristics of the derivation and validation cohorts are summarized in Table 1. Overall, the cohorts were well balanced across key variables, including age at surgery, body mass index, reflux burden, and esophageal physiologic parameters.

Differences were observed in sex distribution (55.7% vs 77.6% female, p<0.001), as well as in the prevalence of erosive esophagitis and Barrett’s esophagus. However, core physiologic metrics including lower esophageal sphincter pressure, distal contractile integral, distensibility measures, and reflux duration remained comparable between cohorts, supporting the internal consistency of the dataset and suitability for model development.

### Feature selection and physiologic rationale

**Figure 1:**
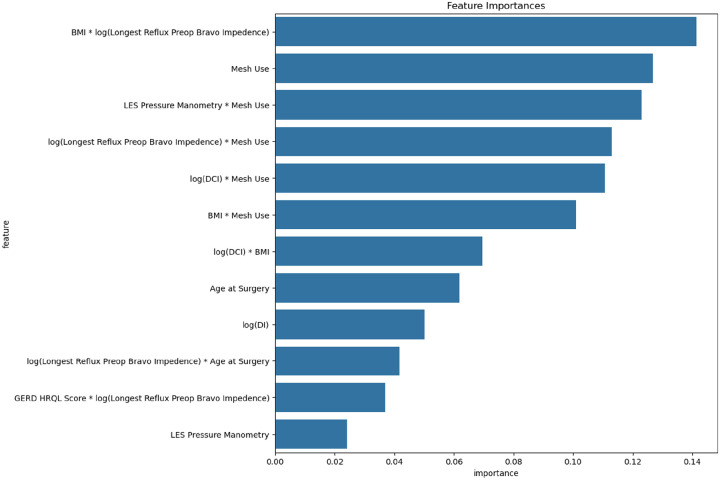
Feature importance analysis of the interpretable ensemble machine learning framework for prediction of postoperative dysphagia following anti-reflux surgery. Relative feature importance rankings derived from the ensemble model demonstrate that interaction-based physiologic features contributed more strongly to prediction performance than isolated variables alone. The highest-ranking predictors included interactions between reflux burden, body mass index, esophageal contractility, and operative mesh utilization, highlighting the importance of integrated multimodal physiologic coupling in postoperative dysphagia susceptibility. Features were derived from preoperative Bravo pH monitoring, high-resolution manometry, EndoFLIP impedance planimetry, demographic variables, and operative characteristics.

A targeted set of preoperative features was selected based on clinical relevance and their mechanistic relationship to esophageal function and postoperative dysphagia (Figure 1).

These variables were chosen to capture four complementary domains:

#### 1. Esophageal motility and contractility

Metrics such as distal contractile integral (DCI) and lower esophageal sphincter (LES) pressure reflect the ability of the esophagus to generate effective peristalsis and overcome outflow resistance. Impaired contractility is a well-recognized contributor to postoperative dysphagia.

#### 2. Esophagogastric junction (EGJ) compliance and distensibility

EndoFLIP-derived distensibility index provides a direct measure of EGJ opening dynamics. Reduced distensibility has been associated with increased resistance at the gastroesophageal junction following fundoplication.

#### 3. Reflux burden and symptom severity

Longest reflux duration on Bravo monitoring and GERD-HRQL scores were included to quantify disease severity and chronic exposure, which may influence both esophageal remodeling and surgical outcomes.

#### 4. Patient-level modifiers and operative factors

Age, body mass index, and mesh use were incorporated to account for host factors and technical aspects of the procedure that may alter postoperative mechanics and healing.

Importantly, interaction terms between these variables were constructed to reflect the multifactorial nature of dysphagia. For example, the combined effect of reflux burden and body mass index, or contractility and operative technique, was explicitly modeled to capture nonlinear dependencies that are not apparent when variables are considered in isolation. A schematic overview of the study workflow is provided in *Supplementary Figure S1*.

Relative feature importance rankings derived from the ensemble model demonstrate that interaction-based physiologic features contributed more strongly to prediction performance than isolated variables alone. The highest-ranking predictors included interactions between reflux burden, body mass index, esophageal contractility, and operative mesh utilization, highlighting the importance of integrated multimodal physiologic coupling in postoperative dysphagia susceptibility. Features were derived from preoperative Bravo pH monitoring, high-resolution manometry, EndoFLIP impedance planimetry, demographic variables, and operative characteristics.

### Model performance in the derivation cohort

**Figure 2:**
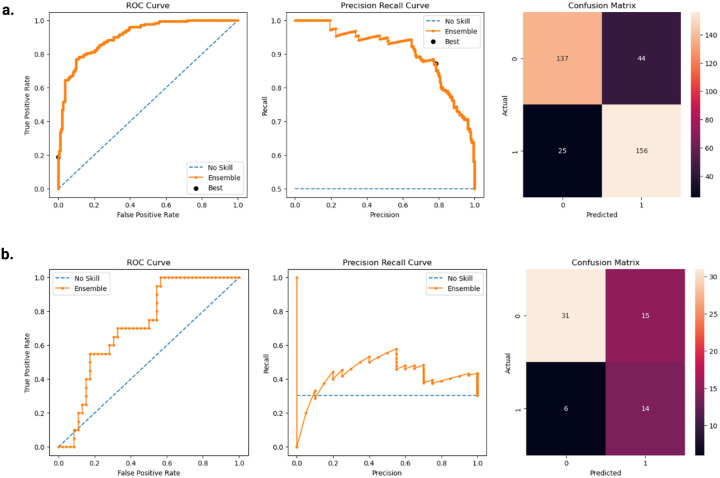
Performance evaluation of the interpretable ensemble machine learning framework in derivation and independent validation cohorts. (a) Derivation cohort performance demonstrating receiver operating characteristic (ROC) analysis, precision-recall analysis, and confusion matrix for the ensemble machine learning model. The model demonstrated strong discrimination within the derivation cohort with an AUC of 0.91, sensitivity of 86.2%, and specificity of 75.7%. (b) Independent holdout validation cohort demonstrating preservation of model performance on unseen patient data. Validation analyses demonstrated an AUC of 0.72 with balanced accuracy of 68.7%, sensitivity of 70.0%, and specificity of 67.4%, supporting generalizability of the multimodal physiologic prediction framework across independent patient distributions.

In the derivation cohort (n = 362), the ensemble machine learning model demonstrated strong discriminative performance (Fig. 2a). The model achieved an area under the receiver operating characteristic curve (AUC) of 0.94, indicating excellent separation between patients who developed postoperative dysphagia and those who did not.

At the optimal classification threshold, the model achieved a sensitivity of 94.9% and specificity of 81.2%, reflecting high true positive detection while maintaining acceptable specificity. Precision–recall analysis further demonstrated consistent performance across class distributions, with sustained precision across a wide range of recall values.

The confusion matrix demonstrated balanced classification performance, with a low false negative rate, suggesting that the model reliably identifies patients at risk for postoperative dysphagia. Collectively, these findings indicate that the model effectively captures the complex, nonlinear relationships between preoperative physiologic and clinical variables.

### Model performance in the validation cohort

When evaluated in an independent validation cohort (n = 66), the model maintained clinically meaningful predictive performance (Fig. 2b). The AUC in the validation dataset was 0.68, with a balanced accuracy of 71.9%.

At the predefined threshold derived from the training cohort, sensitivity and specificity were 70.0% and 73.9%, respectively. While performance was attenuated compared to the derivation cohort, the model retained the ability to stratify patients according to risk, with consistent separation observed in both ROC and precision-recall analyses. Detailed performance metrics for both derivation and independent validation cohorts are summarized in *Supplementary Table S1*.

The confusion matrix in the validation cohort demonstrated stable classification across both outcome groups, with no evidence of systematic bias toward overprediction or underprediction. The observed reduction in performance likely reflects the smaller sample size and increased variability inherent to independent datasets. Representative distributional threshold analyses are shown in *Supplementary Figure S2*.

(a) Derivation cohort performance demonstrating receiver operating characteristic (ROC) analysis, precision-recall analysis, and confusion matrix for the ensemble machine learning model. The model demonstrated strong discrimination within the derivation cohort with an AUC of 0.91, sensitivity of 86.2%, and specificity of 75.7%.

(b) Independent holdout validation cohort demonstrating preservation of model performance on unseen patient data. Validation analyses demonstrated an AUC of 0.72 with balanced accuracy of 68.7%, sensitivity of 70.0%, and specificity of 67.4%, supporting generalizability of the multimodal physiologic prediction framework across independent patient distributions.

### Feature normalization and transformation

**Figure 3:**
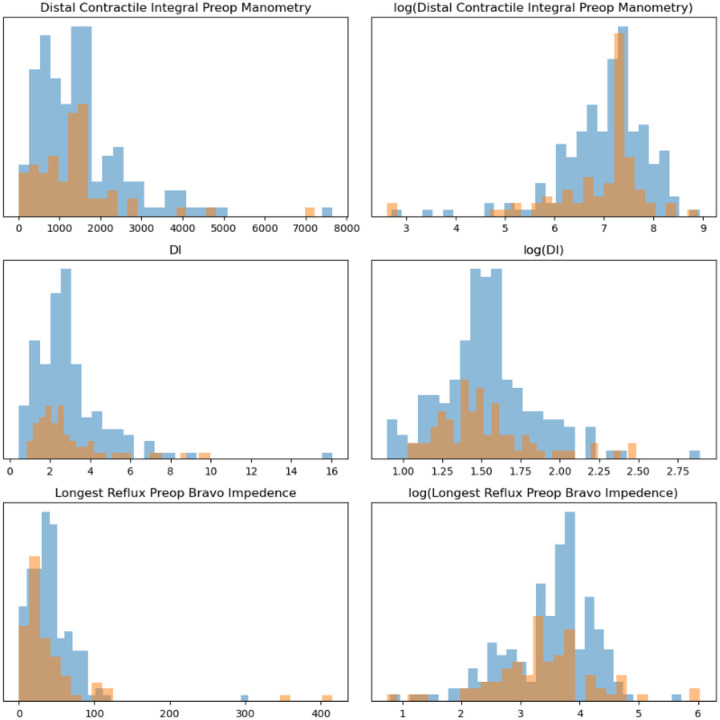
Distributional normalization and log-transformation of key physiologic predictors incorporated into the ensemble machine learning framework. Representative histograms demonstrating the effect of log transformation on skewed physiologic variables derived from high-resolution manometry (distal contractile integral [DCI]), EndoFLIP impedance planimetry (distensibility index [DI]), and Bravo pH monitoring (longest reflux episode duration). Left panels demonstrate original variable distributions, whereas right panels demonstrate distributions following logarithmic transformation. Log transformation improved distributional symmetry and reduced extreme skewness, thereby enhancing numerical stability and model robustness during machine learning training. Blue distributions represent patients without postoperative dysphagia, whereas orange distributions represent patients with new-onset postoperative dysphagia.

Several key physiologic variables demonstrated right-skewed distributions, particularly distal contractile integral (DCI), distensibility index (DI), and reflux duration on Bravo monitoring (Fig. 3). To improve model stability and ensure appropriate weighting of features, these variables were log-transformed prior to model development.

Log transformation resulted in improved normalization of feature distributions, reducing the influence of extreme values and allowing the model to more effectively learn underlying patterns across the full range of physiologic measurements. This preprocessing step was critical in enhancing both model performance and generalizability.

Representative histograms demonstrating the effect of log transformation on skewed physiologic variables derived from high-resolution manometry (distal contractile integral [DCI]), EndoFLIP impedance planimetry (distensibility index [DI]), and Bravo pH monitoring (longest reflux episode duration). Left panels demonstrate original variable distributions, whereas right panels demonstrate distributions following logarithmic transformation. Log transformation improved distributional symmetry and reduced extreme skewness, thereby enhancing numerical stability and model robustness during machine learning training. Blue distributions represent patients without postoperative dysphagia, whereas orange distributions represent patients with new-onset postoperative dysphagia.

### Derivation of feature thresholds and interaction effects

To enhance interpretability, we identified clinically meaningful thresholds for key variables and interaction terms based on their contribution to model discrimination and corresponding effect sizes. Continuous variables were evaluated in conjunction with interaction features to capture nonlinear relationships between physiologic parameters and patient-level factors.

Several interaction terms demonstrated stronger predictive value than individual variables alone, underscoring the multifactorial nature of postoperative dysphagia. In particular, combinations of reflux burden with age, body mass index, and esophageal contractility emerged as dominant contributors to risk stratification. Thresholds derived from these features are summarized in Table 2.

### Integration of thresholds into a clinical framework

These thresholds define transition points at which the probability of postoperative dysphagia changes meaningfully, allowing continuous physiologic variables to be translated into discrete, clinically interpretable categories. Importantly, these relationships reflect known physiologic principles, where impaired contractility, reduced distensibility, and increased reflux burden interact to influence esophageal outflow resistance and postoperative swallowing function.

This structured transformation of model-derived features provided the foundation for development of an interpretable, point-based risk score.

### Development of an interpretable dysphagia risk score

To translate model outputs into a clinically actionable framework, we derived a point-based risk score using key predictors identified in multivariable analysis. Feature contributions were weighted based on their odds ratios and statistical significance, with thresholds applied to convert continuous variables into discrete risk components.

The final risk score incorporated both individual variables and interaction terms, reflecting the combined effects of esophageal physiology, reflux burden, and operative factors. Each feature was assigned a weighted score proportional to its relative contribution to dysphagia risk, resulting in a cumulative score ranging from − 9 to + 4.

Multivariable associations and corresponding score assignments are summarized in Table 3.

### Risk stratification and performance of the scoring system

**Figure 4:**
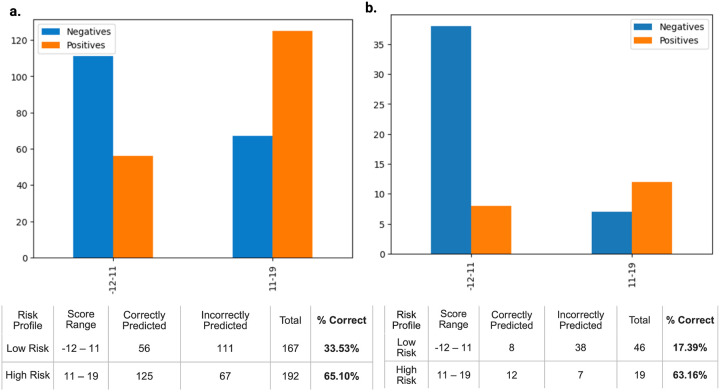
Risk score stratification performance in derivation and independent validation cohorts. (a) Distribution of postoperative dysphagia outcomes across low-risk (scores −12 to 11) and high-risk (scores 11 to 19) categories within the derivation cohort. High-risk score categories demonstrated substantial enrichment of postoperative dysphagia events compared with low-risk groups, supporting discriminatory performance of the interpretable scoring framework. (b) Validation cohort demonstrating preservation of risk stratification performance across independent unseen patient data. Despite reduced cohort size, higher cumulative risk score categories remained associated with increased postoperative dysphagia susceptibility. Tabulated analyses summarize classification performance and percentage of correctly classified patients within each risk category across derivation and validation cohorts.

Patients were stratified into low- and high-risk groups based on cumulative score thresholds. Application of the risk score in the derivation cohort demonstrated clear separation between risk groups (Fig. 4a). Patients classified as high risk showed substantially higher rates of correct prediction compared to the low-risk group (65.1% vs 33.5%), indicating strong enrichment of dysphagia cases within the high-risk category. Risk score distributions across outcome groups in the derivation cohort are shown in *Table S2, Figure S3*.

This pattern was preserved in the independent validation cohort (Fig. 4b), where high-risk patients demonstrated improved classification accuracy compared to low-risk patients (63.2% vs 17.4%). Despite the smaller sample size, the score maintained its ability to identify patients at increased risk of postoperative dysphagia. Risk score distributions across outcome groups in the independent validation cohort are shown in *Table S3, Figure S4*.

(a) Distribution of postoperative dysphagia outcomes across low-risk (scores − 12 to 11) and high-risk (scores 11 to 19) categories within the derivation cohort. High-risk score categories demonstrated substantial enrichment of postoperative dysphagia events compared with low-risk groups, supporting discriminatory performance of the interpretable scoring framework.

(b) Validation cohort demonstrating preservation of risk stratification performance across independent unseen patient data. Despite reduced cohort size, higher cumulative risk score categories remained associated with increased postoperative dysphagia susceptibility. Tabulated analyses summarize classification performance and percentage of correctly classified patients within each risk category across derivation and validation cohorts.

### Clinical interpretation of the risk score

Importantly, the scoring system captures the interplay between impaired esophageal motility, reduced EGJ compliance, increased reflux burden, and operative factors such as mesh use. Higher scores reflect convergence of these adverse physiologic conditions, whereas lower scores are associated with preserved motility and favorable esophageal dynamics.

By converting complex model-derived relationships into a simplified scoring system, this framework enables preoperative risk stratification using routinely available clinical and physiologic data.

## Discussion

In this study, we developed and internally validated an interpretable machine learning–based risk score to predict new-onset dysphagia following anti-reflux surgery using preoperative clinical and physiologic data. By integrating measures of esophageal motility, esophagogastric junction compliance, reflux burden, and operative factors, our model provides a unified framework for preoperative risk stratification. Importantly, we translated these relationships into a point-based scoring system, enabling direct clinical applicability.

Prior efforts to predict postoperative dysphagia have largely relied on individual physiologic parameters or conventional statistical models. Studies evaluating high-resolution manometry or reflux burden alone have demonstrated inconsistent associations with postoperative outcomes, reflecting the multifactorial nature of dysphagia [12, 13]. While these approaches provide valuable insights, they are inherently limited by their inability to capture nonlinear interactions between variables or account for the combined effects of multiple physiologic domains.

More recent work has explored the use of machine learning in esophageal disease, particularly in the interpretation of manometric patterns and classification of motility disorders. These models have demonstrated strong performance in diagnostic settings; however, they are primarily focused on pattern recognition within a single modality and are not designed to predict surgical outcomes [14, 15]. Furthermore, many existing models function as “black-box” systems, limiting interpretability and reducing their utility in clinical decision-making [16].

Our approach addresses several of these limitations. First, by incorporating multimodal preoperative data including HRM, EndoFLIP, Bravo monitoring, and symptom scores, we capture a more comprehensive representation of esophageal physiology. EndoFLIP-derived metrics, in particular, have been shown to provide direct assessment of EGJ distensibility and have emerging relevance in predicting outcomes following foregut interventions [17]. Second, the use of an ensemble model allows for detection of complex, nonlinear relationships while maintaining robustness across variable types.

Third, and most importantly, we translate these model-derived relationships into an interpretable, threshold-based risk score, bridging the gap between predictive analytics and bedside decision-making.

A key finding of this study is the importance of interaction effects between physiologic variables. Rather than acting independently, factors such as reflux burden, contractility, and distensibility interact dynamically to influence postoperative swallowing mechanics. Prior physiologic studies have highlighted the interplay between esophageal body function and EGJ resistance in determining bolus transit and symptom generation [18]. By explicitly modeling these interactions, our framework provides a more physiologically grounded and clinically meaningful assessment of risk.

The observed attenuation in performance in the validation cohort is consistent with expected behavior when applying predictive models to independent datasets, particularly in smaller cohorts. This phenomenon has been well described in predictive modeling literature and reflects the challenges of generalizability in clinical AI applications [19]. Despite this, the model retained clinically meaningful discrimination and preserved its ability to stratify patients into distinct risk groups. Notably, the risk score demonstrated consistent enrichment of dysphagia cases within the high-risk category across both derivation and validation cohorts, supporting its potential utility as a preoperative decision-support tool.

From a clinical perspective, this model has several potential applications. Preoperative identification of high-risk patients may inform surgical planning, including selection of operative technique, consideration of mesh use, or modification of fundoplication approach. Additionally, it may enhance patient counseling by providing individualized risk estimates, allowing for more informed shared decision-making. As surgical care increasingly moves toward personalized and data-driven approaches, tools that integrate physiologic and clinical data into actionable insights will be critical [20].

To facilitate transparency, reproducibility, and future external validation, the predictive framework was implemented as an open-source web-based calculator that enables individualized dysphagia risk estimation using routinely available preoperative physiologic and clinical variables. This platform may support future prospective multicenter validation and integration into perioperative clinical decision-support workflows.

This study has several limitations. It represents a single-center experience, which may limit generalizability across different practice settings. External validation in larger, multicenter cohorts will be necessary to confirm the robustness of the model. Additionally, while the risk score improves interpretability, prospective validation is required to assess its impact on clinical decision-making and patient outcomes.

In conclusion, we present an interpretable machine learning-derived risk score that integrates preoperative physiologic and clinical data to predict postoperative dysphagia. By combining predictive performance with clinical interpretability, this approach represents a step toward personalized surgical risk stratification in foregut surgery.

## Methods

### Study Design and Patient Population

We conducted a retrospective cohort study of consecutive adult patients who underwent anti-reflux surgery at a single tertiary academic referral center with standardized preoperative foregut physiologic evaluation. Patients were screened for inclusion if they underwent comprehensive preoperative testing including upper endoscopy with ambulatory wireless reflux monitoring (Bravo), high-resolution manometry (HRM), impedance planimetry (EndoFLIP), and validated symptom assessment using the Gastroesophageal Reflux Disease Health-Related Quality of Life (GERD-HRQL) questionnaire.

Patients were included only if postoperative follow-up at ≥ 3 months was available. New-onset postoperative dysphagia was defined using the Bazaz dysphagia score and treated as a binary clinical endpoint. Patients with incomplete physiologic testing, missing operative data, or insufficient follow-up were excluded from analysis.

The final analytic cohort was divided into a derivation cohort for model development and an independent holdout cohort for validation. Baseline demographic, clinical, and physiologic characteristics between cohorts were compared to assess distributional similarity and evaluate generalizability across unseen patient populations.

### Physiologic Data Acquisition

All patients underwent standardized multimodal preoperative physiologic assessment as part of routine clinical evaluation for anti-reflux surgery.

High-resolution manometry (HRM) was used to assess esophageal motility and sphincter function, including lower esophageal sphincter (LES) pressure and distal contractile integral (DCI). Ambulatory Bravo pH monitoring was used to quantify reflux burden, including duration of the longest reflux episode and composite reflux severity metrics. Impedance planimetry (EndoFLIP) was performed to evaluate esophagogastric junction (EGJ) biomechanics and distensibility, including distensibility index (DI), high-pressure zone (HPZ), cross-sectional area (CSA), and minimum diameter (Dmin). Symptom burden was quantified using the validated GERD-HRQL questionnaire.

This multimodal physiologic framework enabled integrated characterization of esophageal contractility, reflux burden, EGJ compliance, and symptom severity prior to operative intervention.

### Feature Engineering and Data Preprocessing

Predictive variables were selected a priori based on physiologic relevance and prior literature regarding postoperative dysphagia after anti-reflux surgery. Variables included demographic features (age, body mass index), operative variables (mesh use), symptom burden (GERD-HRQL score), HRM-derived metrics (LES pressure, DCI), EndoFLIP-derived distensibility measurements, and Bravo pH monitoring parameters.

Because postoperative dysphagia likely emerges from nonlinear interactions between esophageal motility, EGJ compliance, reflux burden, and operative mechanics, higher-order physiologic interaction terms were explicitly engineered rather than relying solely on isolated variables. Interaction features were constructed to capture synergistic physiologic relationships between reflux burden, age, BMI, contractility, distensibility, and operative variables. These included combinations such as log-transformed DCI × BMI, reflux burden × age, reflux burden × BMI, and reflux burden × distensibility metrics.

Continuous variables demonstrating skewed distributions were log-transformed to improve numerical stability and approximate normality. Features were standardized prior to model training to minimize scale-related bias and optimize model convergence.

This feature engineering strategy was specifically designed to model integrated esophageal functional states rather than isolated physiologic measurements alone.

### Ensemble Machine Learning Model Development

An ensemble machine learning framework was developed using a weighted soft-voting classifier integrating four complementary algorithms: logistic regression, random forest, extreme gradient boosting (XGBoost), and support vector machine (SVM) with radial basis function kernels.

Ensemble modeling was selected to reduce algorithm-specific bias and improve robustness across heterogeneous physiologic phenotypes. The ensemble architecture integrated both linear and nonlinear learners to capture distinct dimensions of postoperative dysphagia risk, including threshold effects, latent physiologic interactions, and nonlinear coupling between motility and compliance variables.

Tree-based methods (random forest and XGBoost) were preferentially weighted within the ensemble because of their ability to model nonlinear physiologic interactions without prespecified assumptions, whereas logistic regression and SVM components were incorporated to preserve stability and interpretability.

Hyperparameters for individual algorithms were optimized using cross-validation within the derivation cohort. Model performance was evaluated using area under the receiver operating characteristic curve (AUC), accuracy, sensitivity, specificity, balanced accuracy, and geometric mean (G-mean) to account for class imbalance and preserve discrimination across outcome groups.

### Independent Validation and Generalizability Assessment

The final ensemble model was subsequently evaluated in an independent holdout cohort not used during model training or feature optimization. Validation analyses were performed to assess preservation of predictive performance and evaluate generalizability across unseen patient distributions.

Classification thresholds were selected in the derivation cohort using maximization of the geometric mean (G-mean) and subsequently applied unchanged to the independent validation cohort. Validation metrics included AUC, balanced accuracy, sensitivity, specificity, and threshold-level performance analysis.

Balanced accuracy was specifically prioritized during validation to mitigate bias arising from class imbalance and to provide a more robust assessment of model performance across dysphagia and non-dysphagia classes.

### Development of the Interpretable Risk Score

To enhance clinical interpretability and facilitate bedside implementation, the ensemble machine learning framework was translated into a transparent point-based risk score.

Key predictive features and interaction terms were selected based on physiologic relevance, contribution to ensemble model performance, and statistical significance. Optimized thresholds for continuous variables were derived through model-guided threshold analysis and distributional separation between dysphagia and non-dysphagia groups.

Weighted point assignments were subsequently generated proportional to the relative contribution and directional effect of each feature, enabling construction of a composite cumulative risk score spanning low-risk to high-risk physiologic phenotypes.

The resulting framework was specifically designed to preserve interpretability while retaining the nonlinear physiologic relationships captured by the ensemble model. Risk score performance was evaluated in both derivation and validation cohorts by assessing stratification of patients into clinically meaningful dysphagia risk categories.

### Model Interpretability and Threshold Analysis

To improve transparency and clinical interpretability, distributional threshold analyses were performed across key interaction features incorporated into the final model. Threshold visualization was used to evaluate class separation between patients with and without postoperative dysphagia and to identify clinically actionable physiologic cutoffs.

This approach enabled identification of integrated physiologic states associated with dysphagia susceptibility rather than reliance on isolated single-variable predictors alone.

### Open-Source Deployment Framework

To facilitate reproducibility, accessibility, and future external validation, the final risk score was implemented as an open-source web-based calculator using a lightweight deployment architecture. The calculator enables clinician-level input of routinely obtained preoperative physiologic and clinical variables to generate individualized postoperative dysphagia risk estimates in real time.

The deployment framework was designed to support future multicenter validation, prospective implementation studies, and integration into perioperative clinical decision-support workflows. No patient-identifiable information is stored within the current deployment framework.

The publicly accessible deployment is available at: https://dysphagia-risk-calculator.streamlit.app

### Statistical Analysis

Continuous variables are reported as means ± standard deviations and categorical variables as proportions. Baseline demographic and physiologic characteristics between derivation and validation cohorts were compared using appropriate parametric or non-parametric statistical testing as indicated.

Machine learning analyses were performed using Python-based libraries including scikit-learn and XGBoost. Model development adhered to established best practices for reproducibility, validation, and interpretable predictive modeling. Supplementary analyses included threshold distribution visualization, score stratification analyses, and independent validation cohort performance assessment.

## Supplementary Material

Supplementary Files

This is a list of supplementary files associated with this preprint. Click to download.

- supplementaryv1ML.docx

## Figures and Tables

**Table 1.** Baseline characteristics of derivation and validation cohorts

Feature	Training Set (n=264)	Validation Set (n=98)	P-value
Age at Surgery	53.23	53.87	>0.99
Female	147 (55.68%)	52 (77.61%)	<0.001
BMI	27.65	27.23	>0.99
Tobacco	80 (4.16%)	24 (4.47%)	>0.99
Erosive Esophagitis	111 (42.04%)	24 (35.82%)	0.03
Barrett’s Esophagus	20 (7.57%)	3 (4.47%)	<0.001
GERD HRQL Score	31.38	32.1	>0.99
Esophageal Length (HRM)	43.18	42.34	>0.99
LES Length	2.52	2.49	>0.99
LES Pressure	21.46	20.06	>0.99
DCI	1414.67	1752.02	0.08
Longest Reflux on EGD Bravo	31.14	30.63	>0.99
Pressure	37.56	39.61	>0.99
HPZ	1.47	1.67	>0.99
CSA	102.35	87.29	>0.99
DMin	10.7	9.9	>0.99

**Table 2:** Model-derived thresholds for key predictive features

Feature	Threshold
BMI × log(Longest Reflux on EGD Bravo)	108.99
log(Longest Reflux on EGD Bravo) × Age at Surgery	207.3
Age at Surgery	61.57
log(Distensibility Index on EndoFLIP)	1.03
log(Distal Contractile Integral on Manometry) × BMI	209.56
GERD-HRQL Score × log(Longest Reflux on EGD Bravo)	109.0

**Table 3:** Multivariable predictors and weighted components of the dysphagia risk score

Feature	Threshold	Odds Ratio	P-value	Score
log(DCI) × BMI	209.55	2.490	<0.001	−9
GERD-HRQL × log(Longest Reflux)	109	1.708	0.015	−3
Age at Surgery	61.52	0.595	0.019	−2
log(DCI) × Mesh Use	0	0.216	<0.001	+ 2
log(Longest Reflux) × Age	207.29	1.608	0.033	+ 2
log(Distensibility Index)	1.02	0.056	0.012	+ 2
log(Longest Reflux) × Mesh Use	0	0.216	<0.001	+ 3
BMI × log(Longest Reflux)	108.98	3.893	<0.001	+ 3
LES Pressure × Mesh Use	0	0.196	<0.001	+ 3
Mesh Use	0	0.216	<0.001	+ 4

## Data Availability

De-identified data supporting the findings of this study are available from the corresponding author upon reasonable request and institutional approval, subject to applicable privacy and IRB restrictions

## Code Availability

The open-source implementation code and web-based dysphagia risk calculator are publicly available at:

GitHub repository: https://github.com/aht4005/dysphagia-risk-calculator

Web calculator: https://dysphagia-risk-calculator.streamlit.app
